# Neighbourhood deprivation, smoking, and race in South Africa: A cross-sectional analysis

**DOI:** 10.1016/j.pmedr.2018.07.001

**Published:** 2018-07-04

**Authors:** Yan Kwan Lau, Jamie Tam, Nancy L. Fleischer, Rafael Meza

**Affiliations:** a Department of Epidemiology, School of Public Health, University of Michigan, Ann Arbor, MI 48109, 1415 Washington Heights, United States of America; b Department of Health Management and Policy, School of Public Health, University of Michigan, Ann Arbor, 1415 Washington Heights, United States of America; c Center for Social Epidemiology and Population Health, School of Public Health, University of Michigan, 1415 Washington Heights, Ann Arbor, United States of America

**Keywords:** Smoking, South Africa, Residential characteristics, Epidemiology

## Abstract

Research on the role of neighbourhood-level deprivation in low- and middle-income countries with respect to tobacco use is relatively nascent. In South Africa, where race and deprivation are closely linked due to the history of apartheid, smoking disparities exist by individual risk factors such as gender, race, and socioeconomic status. However, less is known about how community-level factors affect smoking disparities in the country, or how the relationship between deprivation and smoking differs by race. We used data from the 2008 South African National Income Dynamics Study (NIDS) and Poisson generalised estimating equations to assess the relationship between neighbourhood deprivation and current smoking for individuals nested within neighbourhoods, while controlling for individual-level and household-level covariates. Subgroup analyses for racial categories Black and Coloured were performed. We found that the relationship between neighbourhood deprivation and smoking prevalence was non-linear: the smoking prevalence ratio was highest among those in the middle range for our deprivation index, and lower at extremely high and low levels of deprivation. Both Black and Coloured subsamples exhibited this inverted U-shape, although the relationship was weaker in the latter group. That the relationship between neighbourhood deprivation and smoking is non-linear contrasts with what has been found in high-income countries, where the relationship between neighbourhood deprivation and smoking is linear. Moreover, these findings are relevant to assess the potential differential impact of smoking interventions as a function of socioeconomic and environmental context.

## 1 Introduction

South Africa is an upper-middle income country with a population of 55.9 million in 2016 (Statistics South Africa, 2016). Most of the population is Black (80.7%), followed by Coloured (8.8%; ethnic classification of persons with mixed ancestry), White (8.1%), and Indian/Asian (2.5%) (Statistics South Africa, 2016). The policy of apartheid in South Africa from 1948 to 1994 enforced segregation with the most resources allocated to Whites and the least to Blacks (Posel, 1991). Coloured and Asian/Indian groups were also subject to discriminatory practices that gave preferential treatment to Whites; however, they were conferred social and economic advantages over Blacks (Erasmus, 2001). These practices produced extreme disparities in education, employment, housing, living conditions, access to healthcare, and health outcomes along racial lines, which continue today (Kon and Lackan, 2008; Moller, 1998). In fact, South Africa is ranked as one of the most unequal nations in the world (Central Intelligence Agency (CIA), 2016).

While smoking prevalence has declined nationally from 30.2% in 1995 to 17.6% in 2012 (Reddy et al., 2015; Reddy et al., 2013; van Walbeek, 2004), disparities by race have persisted despite South Africa's implementation of progressive tobacco control policies, including: the Tobacco Products Control Act in 1993, the ratification of the WHO Framework Convention on Tobacco Control in 2005, and amendments to the original act in 2007 and 2008 (Reddy et al., 2013; van Walbeek, 2004). The 2012 South African National Health And Nutrition Survey reported that men having a considerably higher smoking prevalence (29.2%) than women (7.3%) (Reddy et al., 2015). In addition to gender, smoking disparities based on race, socioeconomic status, and geographic location (urban/rural), are also present. For example, Coloured men have a much higher smoking prevalence than other racial groups: 47.0% compared to 28.5% among Black men, 18.0% among White men, and 36.8% among Asian/Indian men (Reddy et al., 2015). Similarly, Coloured women have the highest smoking prevalence with 34.4%, followed by 12.9% among White women, 7.5% among Asian/Indian women, and 3.3% among Black women (Reddy et al., 2015). Corresponding disparities in lung cancer mortality have persisted since the 1970s, where Coloured men die disproportionately more from lung cancer than any other group (Bradshaw and Harington, 1975; Sitas et al., 2013).

Although several individual-level predictors for adult smoking behaviours in South Africa have been established (Peer et al., 2009; Reddy et al., 2015; Strebel et al., 1989; Vellios and van Walbeek, 2016), less is known about how community-level factors affect smoking disparities in the country. Neighbourhood environments can contribute to health disparities through pathways that involve the physical environment, local institutions, cultural norms, and behavioral mediators related to stress (Diez Roux and Mair, 2010). As one marker of the neighbourhood environment, neighbourhood deprivation might encourage smoking through social norms, lack of institutional resources to support healthy decisions, or weak enforcement of existing tobacco control and other health regulations (Diez Roux and Mair, 2010). Previous research in high-income countries (HICs) has shown that neighbourhood economic and social deprivation are associated with higher levels of tobacco use, including higher smoking prevalence and earlier ages of smoking initiation (Baumann et al., 2007; Blakely et al., 2014; Duncan et al., 1999; Kleinschmidt et al., 1995; Lakshman et al., 2011). However, to our knowledge only one study has explored the relationship between neighbourhood deprivation on smoking behaviours in a low- or middle-income nation (Fleischer et al., 2015). In contrast to findings from the literature on HICs, Fleischer et al. found that higher levels of neighbourhood deprivation in Mexico was associated with better smoking outcomes, such as lower smoking intensity and increased number of quit attempts (Fleischer et al., 2015). The extent to which this pattern of higher deprivation and better smoking outcomes is true in the context of sub-Saharan Africa is unknown. Sub-Saharan Africa is an important area for tobacco control intervention because smoking rates are still quite low in the region overall. Such nations are in the early stages of the tobacco epidemic, but are expected to experience substantial increases in smoking due to increased marketing by tobacco companies and increased affordability of cigarettes with rising incomes (Blecher, 2010; Blecher and Ross, 2013; Blecher and van Walbeek, 2004). The case of South Africa is especially worthy of examination because of stark differences in neighbourhood environments by race (Gradín, 2012).

Using data from the National Income Dynamics Study (NIDS) (Southern Africa Labour and Development Research Unit, 2016), we conducted the first analysis of the relationship between neighbourhood-level deprivation and smoking in South Africa. We used the first wave of NIDS from 2008 as this was around the same time when the amendments of the Tobacco Products Control Act took place in response to the ratification of the FCTC (Reddy et al., 2013; van Walbeek, 2004); that is, we could reasonably study the association of neighbourhood deprivation and smoking prior to significant changes in the tobacco control policy environment. We hypothesised that higher neighbourhood deprivation would be associated with increased smoking, regardless of race.

## 2 Materials and methods

### 2.1 Population

NIDS is a nationally representative panel study of South Africa conducted biennially by the Southern Africa Labour and Development Research Unit. The survey assesses population demographics, levels of education, income dynamics, health, well-being, social cohesion, and household socioeconomic status. NIDS used a stratified, two-stage cluster design to sample households included in the base wave in 2008. Data were collected from a nationally representative sample of 7305 households belonging to 400 Primary Sampling Units (PSUs), which were derived from 2001 Census Enumeration Areas (EAs). The PSU is the smallest geographical unit in the NIDS dataset, containing between one to four EAs such that a PSU will have a minimum of 74 households; we use PSUs as a proxy for neighbourhoods. Further details regarding the questionnaire, survey design, and sampling methodology have been described elsewhere (Leibbrandt et al., 2009). Here, we use the 2008 NIDS Adult (ages 15+) and Household questionnaires to provide a baseline description of the possible association between neighbourhood deprivation and smoking.

### 2.2 Smoking status

Current smoking status was determined by a “Yes” or “No” response to the question “Do you smoke cigarettes?” For those who answered “No”, the follow-up question was “Did you ever smoke cigarettes regularly?” where former smokers are those who answered “Yes” and never smokers are those who answered “No”. For our analysis, we excluded former smokers to make the comparison between current smokers and never smokers.

### 2.3 Neighhourhood deprivation

To assess the level of neighbourhood deprivation, we used the validated 2007 South African Index of Multiple Deprivation (SAIMD) (Wright and Noble, 2009). The SAIMD considers four domains: income and material, employment, education, and living environment deprivation (see Table 1 for details on specific measures). We extracted the relevant information from NIDS to compute the SAIMD domain scores for each of the neighbourhoods in our data. We then followed the procedures developed for the SAIMD to combine the domain scores (Noble et al., 2006; [Bibr bb0125][Bibr bb0115]). First, we standardised the domain scores by ranking them, then scaling the ranks to a range between 0 and 1 by R, where *R* = 1/N for the least deprived, and R = N/*N* = 1 for the most deprived neighbourhood. The ranks were then transformed by the following truncated exponential distribution:

$${\mathrm{Score}}_{\mathrm{Domain}}=-\mathrm{\delta ln}\left\{1-R\left[1-e^{-\frac{100}{\delta }}\right]\right\}$$

where δ is a constant that stretches out the distribution such that approximately 25% of the neighbourhoods have a score of 50 or higher (Noble et al., 2013). This transformation ensured that when the scores from the 4 domains were combined, lack of deprivation on one domain would not cancel out presence of deprivation in another. The maximum score for each domain was 100 (i.e. most deprived) while the minimum was zero (least deprived). Finally, the domain scores were summed, each carrying a weight of 0.25. We used this combined measure of deprivation for our analyses.

**Table 1:** Description of deprivation domains.

Deprivation domains	Domain components
Income and material	Number of people living in a household: - with income below 40% of the mean equivalent household income (1167 ZAR/146 USD in 2008), OR - without a refrigerator OR neither a television nor radio ***divided*** by the total number of people in the neighbourhood
Employment	Sum of the number of people who are unemployed and the number of people who are not working due to health reasons in the neighbourhood ***divided*** by the sum of the totally economically active population (aged 18–65) and those not working for health reasons in the neighbourhood
Education	Number of adults aged 18–65 with no secondary education ***divided*** by all adults aged 18–65 in the neighbourhood
Living environment	Number of people in the neighbourhood - Living in a shack, OR - In a crowded household, OR - In a household **without** either piped water inside their dwelling or yard, - OR without a pit latrine with ventilation OR flush toilet, - OR without use of electricity for lighting ***divided*** by the total number of people in the neighbourhood.

### 2.4 Covariates

We controlled for potential factors that could confound the association between neighbourhood deprivation and smoking based on findings from previous studies. Age and age-squared were entered as continuous variables to allow for a non-linear relationship between current smoking and age. Education had three categories: primary school or less (referent), some high school, and completed high school or more. Employment status consisted of unemployed, not economically active (i.e. those who are not employed and who are not seeking employment), and employed (referent). We also included gender and urban/rural area of residence. Household income quintiles were used with the fifth quintile (i.e. wealthiest) as the reference category, and were derived independently within the Black and Coloured subsamples, respectively, due to the stratified nature of our subsequent analysis.

### 2.5 Statistical analysis

We used generalised estimating equations to test for associations between neighbourhood deprivation and individual smoking status, accounting for correlation within neighbourhoods (Hubbard et al., 2010). Poisson models with robust error variance were used instead of logistic models to calculate prevalence ratios, given that our outcome of interest, current smoking, was not rare (Deddens and Petersen, 2008; Zou, 2004).

**Fig. 1:**
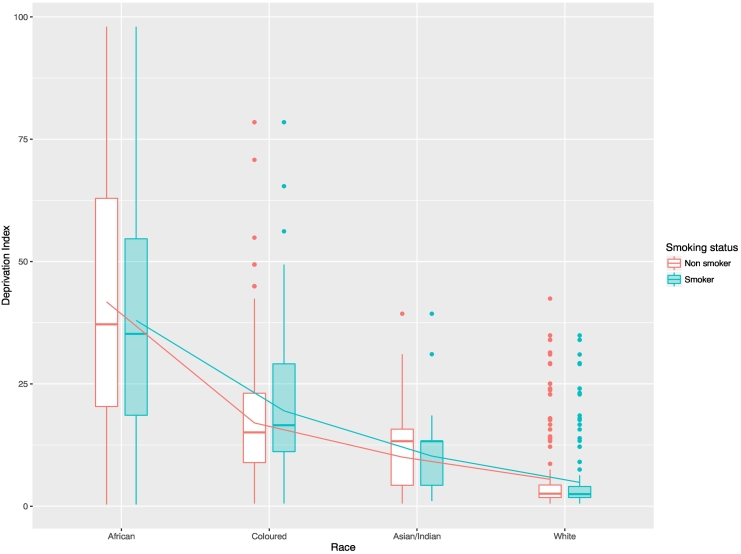
Box plot of neighbourhood deprivation by race and smoking status.

Our analyses were restricted to Black and Coloured populations for the following reasons: 1) only Black and Coloured groups had response rates of over 70% for the survey (Indian/Asian and White had 66% and 36% respectively); 2) the subsample size for Indian/Asian was too small and sampling weights were not reliable for subgroup analyses; and 3) there was little to no variation in levels of neighbourhood deprivation for White and Indian/Asian groups (see Fig. 1), a legacy of apartheid policies. Related to the third reason, we conducted subgroup analyses by race as there would be collinearity if we had entered race and neighbourhood deprivation as covariates together. Hence, we fitted 4 models for Black and Coloured subsamples separately: Model 1 is the unadjusted model with deprivation (linear) as the exposure of interest and current versus never smoking as the outcome; Model 2 adds an additional squared term for neighbourhood deprivation to Model 1 to explore the possibility of a non-linear association with smoking status; Model 3 adjusts the relationship for individual-level variables; Model 4 adds household-level variables onto Model 3. All models accounted for sampling weights, using the post-calibrated weights provided by the NIDS dataset, and models were run with SAS software v.9.4 (SAS Institute, Cary NC). Descriptive statistics were calculated in R 3.3.1 (R Core Team, 2016) with the **survey** package (Lumley, 2004). All figures were created with the R package **ggplot2** (Wickham, 2009).

## 3 Results

Table 2 presents selected characteristics of the study sample. Observations were dropped due to incomplete data on smoking status (*n* = 67 and *n* = 7 for Black and Coloured respectively), and covariates of interest (*n* = 143 and *n* = 49 for Black and Coloured respectively). In the Black subsample (*n* = 12,036), 16.5% were current smokers, compared to 80.4% who were never smokers. 40.2% were employed and just over half (54.6%) lived in urban areas. The average monthly household income was R3873.0 (SE = 220.8), and 57.3% lived in neighbourhoods with medium to high/very high deprivation. In the Coloured subsample (*n* = 2148), 42.0% reported being current smokers (more than twice the proportion reported compared to the Black subsample), compared to 47.4% who were never smokers. The majority lived in urban areas (89.4%) and 51.6% were employed. The average monthly household income was R6696.6 (SE = 686.5), and 21.2% (less than half the proportion reported compared to the Black subsample) lived in neighbourhoods with medium to high/very high deprivation.

**Table 2:** Descriptive characteristics of the sample – Black and Coloured only (South Africa, 2008).

	Black (*N* = 12,036)	Coloured (*N* = 2148)
	n (%, weighted)	
Smoking status		
Current smoker	1847 (16.5%)	1013 (42%)
Former smoker	303 (3.1%)	231 (10.7%)
Never smoker	9886 (80.4%)	904 (47.4%)
Male	4798 (44%)	848 (40.8%)
Age group		
<17	1105 (8.2%)	138 (6.1%)
18–24	2700 (22.8%)	336 (14.4%)
25–34	2426 (24.3%)	407 (24.4%)
35–44	1905 (17.8%)	448 (21.6%)
45–64	2706 (20.8%)	628 (27%)
65+	1194 (6.2%)	191 (6.5%)
Education		
Completed primary school or less	4897 (32.8%)	869 (28.9%)
Some high school	4776 (42.6%)	873 (44.1%)
Completed high school or more	2363 (24.6%)	406 (27.1%)
Employment status		
Employed	4274 (40.2%)	1027 (51.6%)
Unemployed - Discouraged/Strict	2339 (20.5%)	361 (18%)
Not economically active	5423 (39.4%)	760 (30.5%)
Urban	4756 (54.6%)	1711 (89.4%)
Monthly household income^a^	3873.0 (220.8)	6696.6 (686.5)
Neighbourhood deprivation		
Very low	972 (14.6%)	546 (52.6%)
Low	2931 (28.1%)	1031 (26.1%)
Medium	3478 (29.3%)	539 (19.7%)
High/Very high	4655 (28.0%)	32 (1.5%)

a The mean and standard deviation is given for monthly household income in South African Rand (one rand is approximately equivalent to US$0.125 in 2008).

Model 1 and 2 were unadjusted models, with only the linear term for deprivation in the former, and the quadratic term added to the latter. In the Black subsample (Table 3, *n* = 11,733 after excluding former smokers), a non-linear association with current smoking status was apparent from Model 2's estimates (PR_Deprivation_ = 1.0185, 95% CI [1.0082,1.0288]; PR_Deprivation-squared_ = 0.9998, 95% CI [0.9996,0.9999]). However, in the Coloured subsample (Table 4, *n* = 1917 after excluding former smokers), the unadjusted association between deprivation and being a current smoker appeared to be linear, as seen from Model 1's estimates (PR_Deprivation_ = 1.0199, 95% CI [1.0049,1.0189]).

**Table 3:** Results of Poisson models for the Black subsample (*n* = 11733^a^).

	Prevalence ratios (95% CI)			
	Model 1	Model 2	Model 3	Model 4
Deprivation index	0.9980 (0.9956, 1.0004)	1.0185 (1.0082, 1.0288)	1.0156 (1.0059, 1.0253)	1.0146 (1.0046, 1.0247)
(Deprivation)^2^		0.9998 (0.9996, 0.9999)	0.9998 (0.9997, 0.9999)	0.9998 (0.9997, 0.9999)
Age			1.0227 (1.0177, 1.0278)	1.0231 (1.0180, 1.0283)
(Age)^2^			0.999 (0.9987, 0.9993)	0.999 (0.9988, 0.9993)
Male			9.4152 (7.9882, 11.0972)	9.4539 (8.0024, 11.1687)
Primary school or less			1.7286 (1.4557, 2.0526)	1.5917 (1.3294, 1.9058)
Some high school			1.4460 (1.2203, 1.7135)	1.3629 (1.1432, 1.6250)
Unemployed			1.2328 (1.0880, 1.3969)	1.1481 (1.0077, 1.3080)
Not economically active			0.7592 (0.6522, 0.8838)	0.7314 (0.6291, 0.8503)
Urban				1.1492 (1.0108, 1.3066)
Income quintile 1				1.4196 (1.1465, 1.7578)
Income quintile 2				1.3794 (1.1159, 1.7051)
Income quintile 3				1.2806 (1.0406, 1.5759)
Income quintile 4				1.0397 (0.8336, 1.2968)

a Excludes former smokers.

**Table 4:** Results of Poisson models for the Coloured subsample (n = 1917^a^).

	Prevalence ratios (95% CI)			
	Model 1	Model 2	Model 3	Model 4
Deprivation index	1.0119 (1.0049, 1.0189)	1.0266 (1.0035, 1.0503)	1.0184 (0.9952, 1.0421)	1.0092 (0.9828, 1.0363)
Deprivation^2		0.9997 (0.9993, 1.0001)	0.9997 (0.9994, 1.0001)	0.9998 (0.9994, 1.0002)
Age			1.0069 (1.0006, 1.0132)	1.0073 (1.0012, 1.0135)
(Age)^2^			0.9994 (0.9990, 0.9998)	0.9994 (0.9990, 0.9998)
Male			1.4256 (1.211, 1.6782)	1.4014 (1.1883, 1.6526)
Primary school or less			1.8562 (1.3949, 2.4699)	1.6494 (1.2339, 2.2048)
Some high school			1.4766 (1.1232, 1.9413)	1.3403 (1.0189, 1.7631)
Unemployed			1.0395 (0.7835, 1.3791)	0.9822 (0.7431, 1.2982)
Not economically active			0.7509 (0.5986, 0.9419)	0.7408 (0.5918, 0.9272)
Urban				0.8865 (0.7136, 1.1012)
Income quintile 1				1.5537 (0.9732, 2.4804)
Income quintile 2				1.2266 (0.7859, 1.9144)
Income quintile 3				1.4054 (0.9125, 2.1646)
Income quintile 4				1.1973 (0.7633, 1.8781)

a Excludes former smokers.

Model 4 shows the fully adjusted models for the Black and Coloured subsamples, respectively. Neighbourhood deprivation retained a non-linear association with smoking status among Blacks (PR_Deprivation_ = 1.0146, 95% CI [1.0046,1.0247]; PR_Deprivation-squared_ = 0.9998, 95% CI [0.9997,0.9999]). In the Coloured subsample (Table 4), the same models were fitted; however, the non-linear relationship between neighbourhood deprivation and current smoking was attenuated (PR_Deprivation_ = 1.0092, 95% CI [0.9828,1.0363]; PR_Deprivation-squared_ = 0.9998, 95% CI [0.9994,1.0002]). Given the estimates from Model 1, we additionally fitted models adjusted for individual- and household-level covariates for the Coloured subsample without the quadratic deprivation term; however, the prevalence ratio for the linear deprivation term also crossed the null (results not shown).

With regards to other covariates for the Black subsample in Table 3, being male (versus female), having an education less than high school completion (versus more), being unemployed (versus employed), and living in an urban area (versus rural), were found to be associated with a higher prevalence of being a current smoker. Notably, the prevalence of smoking was 9.4 times (95% CI [7.99,11.10]) higher among males compared to females for the Black subsample. Age had a non-linear association with current smoking status, whereas household income showed a monotonically decreasing relationship with being a current smoker. Similar associations were observed for covariates for the Coloured subsample, except for living in an urban area (Table 4; PR = 0.89, 95% CI [0.71,1.10]). Furthermore, the prevalence of smoking was only 1.40 times (95% CI [1.19,1.65]) higher among males compared to females.

**Fig. 2:**
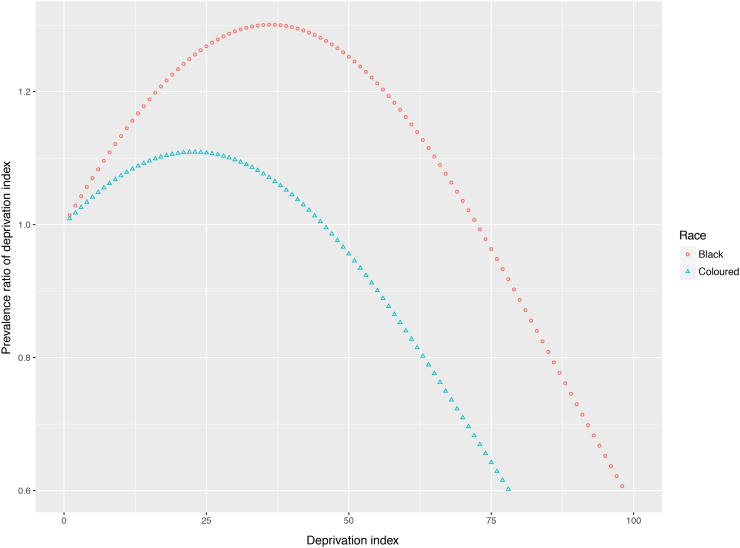
Scatterplot of the association of neighbourhood deprivation and smoking status by race.

Fig. 2 shows the prevalence ratio (vertical axis) across the range of potential values that the deprivation index (horizontal axis) could take. From the inverted U-shape, we see that neither the most nor the least deprived neighbourhoods had the highest prevalence ratio; this occurred in neighbourhoods in between the two extremes of deprivation. For both the Black and Coloured subsamples, the prevalence ratio is lowest at the highest levels of deprivation, where deprivation is most negatively associated with smoking.

## 4 Discussion

This study adds to an emerging literature on community-level factors and health behaviours in developing nations and is the first to examine the association between area deprivation and smoking in a sub-Saharan African context. Using nationally representative data and a validated measure for deprivation, we found that in the context of South Africa, the relationship between neighbourhood deprivation and smoking exhibits differences by race and appears to be non-linear. Our results indicate that the smoking prevalence ratio is highest among those in the middle range for our deprivation index, although this relationship was less marked for the Coloured subsample than the Black subsample. In contrast, studies in HICs that found neighbourhood deprivation to be an independent predictor of smoking status showed a monotonic trend; that is, the higher the neighbourhood-level deprivation, the higher the odds of being a current smoker (Baumann et al., 2007; Blakely et al., 2014; Blecher and van Walbeek, 2004; Fleischer et al., 2015; Lakshman et al., 2011). When comparing our study to studies conducted in HICs, this difference in association between deprivation and smoking may well be due to neighbourhood deprivation in South Africa having a different meaning than in a more developed country. For example, “use of electricity for lighting” is not considered in deprivation indices for developed countries. In other words, a highly deprived neighbourhood in South Africa, particularly a Black neighbourhood, is not necessarily comparable to a highly deprived neighbourhood in the UK. At very high levels of deprivation, the stressors that contribute to an individual's decision to smoke may be very different in South Africa versus HICs. Our results for South Africa are consistent with findings from Mexico, which found highest levels of deprivation to be associated with lower smoking.

In France, Chaix and Chauvin suggested that regional differences in smoking prevalence could be due to different levels of consumerism, levels of advertising and the availability of retail outlets (Chaix and Chauvin, 2003). Even though the South African context is clearly different, these factors may also contribute to our findings. For example, cigarette packs sold by official retailers may be inaccessible in rural neighbourhoods, although in informal settings in South Africa where socioeconomic disadvantage is higher, they are often sold illegally as ‘loosies’ or as a single cigarette rather than by the pack (Wherry et al., 2014). At the lowest levels of deprivation, smoking prevalence may be lower because of social norms that discourages tobacco use. In general, those living in well-resourced neighbourhoods tend to also have greater access to public health and health care infrastructures, along with social norms, that discourage substance use or other unhealthy behaviours (Pampel et al., 2010). Those living in medium-deprivation settings may in contrast have increased access to cigarettes and the means to get them, yet not be influenced by social norms that discourage smoking. Further research is required to understand the underlying mechanisms driving this pattern of tobacco use across the population in South Africa and other developing countries.

We also observed important differences in the relationship between neighbourhood-level deprivation and smoking by race group. The association between deprivation and smoking was less clear for the Coloured population, which has the highest smoking rates in South Africa. Given that the effect size for neighbourhood deprivation was relatively small, a larger sample size may have been required to detect a deviation from null. On the other hand, smoking rates for Coloured individuals may be sufficiently high such that deprivation at the community level would have a limited effect in settings where social norms that promote smoking already exist. At the time of survey, smoking prevalence in the Coloured population (42.0%) was >2.5 times that of the Black population (16.5%). The stronger association between deprivation and current smoking prevalence among Blacks relative to Coloureds suggests that deprivation might have greater impact on smoking behaviours where smoking is less common, as in Black communities, particularly among women where it had been a cultural taboo to smoke (Marks et al., 2001; Peltzer, 2001; Williams et al., 2008). As communities in South Africa develop economically and efforts to reduce economic inequality continue, Blacks, who disproportionately occupy the most deprived segments of society, may shift into environments that put them at greater risk for smoking uptake. Tobacco control efforts in the country must continue to reach all populations, and ensure that Black and female smoking rates do not rise.

In addressing smoking disparities and evaluating the effectiveness of tobacco control strategies, it is crucial to understand the role of area-level influences on smoking-related outcomes. The impact of policies such as smoking cessation programs, smoke-free air legislation, or restrictions on youth purchasing of tobacco products, depends on the local context in which they are implemented. This knowledge for the South African context could be valuable for public health practice, and for the implementation of programs targeting the most at-risk communities.

Since we used cross-sectional data, we are unable to draw conclusions about whether neighbourhood deprivation causes people to smoke, as our exposure, outcome and potential confounders were all measured at the same time point. We also did not consider the length of time a person has lived in his/her neighbourhood, which could affect the association between neighbourhood deprivation and smoking. Additionally, while PSUs are the smallest spatial unit available in the dataset, modifiable areal unit problem could be a source of bias on our findings; i.e. we assume that the PSUs are representative of the exposure to neighbourhood deprivation relevant to smoking status. Finally, our measure of deprivation is an aggregate of individual-level and household-level variables, making it compositional in nature, and thus may not capture structural features of deprivation in a neighbourhood that could be more pertinent to smoking and other health-related behaviours. Nonetheless, this measure captures important aspects of deprivation and was based on a validated index, which has been used by South African policy makers to profile poverty and deprivation across the country (Noble et al., 2013; Noble and Wright, 2013; Wright and Noble, 2009).

## 5 Conclusions

Contrary to studies in HICs, our study found that neighbourhood deprivation had a non-linear relationship with smoking status among a nationally representative sample in South Africa in 2008. The strength of association was modified by race, where we saw a stronger association among the Black compared to the Coloured population. Future studies should explore the possible effects of a change in neighbourhood deprivation on smoking status, smoking intensity, and smoking initiation and cessation in the context of a developing country.
